# Comparison of protective effects of teneligliptin and luseogliflozin on pancreatic β-cell function: randomized, parallel-group, multicenter, open-label study (SECRETE-I study)

**DOI:** 10.3389/fendo.2024.1412553

**Published:** 2024-10-21

**Authors:** Masashi Shimoda, Yukino Katakura, Akiko Mashiko, Masahiro Iwamoto, Shuhei Nakanishi, Takatoshi Anno, Fumiko Kawasaki, Atsushi Obata, Yoshiro Fushimi, Junpei Sanada, Kenji Kohara, Hayato Isobe, Yuichiro Iwamoto, Hidenori Hirukawa, Fuminori Tatsumi, Yukiko Kimura, Tomohiko Kimura, Tomoatsu Mune, Kohei Kaku, Hideaki Kaneto

**Affiliations:** ^1^ Department of Diabetes, Endocrinology and Metabolism, Kawasaki Medical School, Kurashiki, Japan; ^2^ Iwamoto Medical Clinic, Zentsuji, Japan; ^3^ Department of General Internal Medicine 1, Kawasaki Medical School, Okayama, Japan

**Keywords:** β-cell function, DPP-4 inhibitor, proinsulin, SGLT2 inhibitor, type 2 diabetes

## Abstract

**Aims:**

The aim of this study is to directly compare the effects of SGLT2 inhibitors and DPP-4 inhibitors on β-cell function in patients with type 2 diabetes.

**Materials and methods:**

We conducted a 26-week, randomized, open-label, parallel-group study, including a 1-2 week drug washout period, in patients with type 2 diabetes with HbA1c ≥7.0% and <9.0% and BMI ≥20 kg/m^2^ despite treatment with a drug naïve or other than DPP-4 inhibitors or SGLT2 inhibitors. A total of 103 subjects were randomly assigned to receive once daily oral luseogliflozin (L) or teneligliptin (T). The primary endpoint was the effect of L vs. T on the change in logarithmus naturalis (Ln) disposition index (DI) (DI _0-120min_; combining measures of insulin secretion and sensitivity) from baseline to week 25-26 (post intervention), which was calculated by conducting an oral glucose tolerance test.

**Results:**

Ln DI _0-120min_ were improved in both groups: -0.46 ± 0.68 to -0.20 ± 0.59 (*p*=0.03) in L group and -0.26 ± 0.60 to -0.05 ± 0.62 (*p*=0.01) in T group. The change in Ln serum proinsulin/C-peptide ratio, a marker of β-cell dysfunction, was reduced in L group (1.63 ± 0.63 to 1.56 ± 0.68, *p*=0.16), but rather increased in T group (1.70 ± 0.75 to 1.90 ± 0.51, *p*=0.01), with significant difference between the two groups (-0.27; *p*=0.004).

**Conclusions:**

Improvement of disposition index in subjects with obese type 2 diabetes was comparable between luseogliflozin and teneligliptin. On the other hand, it is likely that alleviation of β-cell dysfunction is more effective with luseogliflozin compared to tenegliptin.

**Clinical trial registration:**

https://rctportal.niph.go.jp/en, identifier jRCTs061190008.

## 1 Introduction

The number of patients with type 2 diabetes remains a major social and medical problem (1, 2). The major complications such as macro- and microangiopathy impair healthy life expectancy and quality of life (3, 4), and increase the risk of death (5). The strict glycemic control suppressed the onset and progression of microangiopathy (6) and reduced the risk of death and ischemic heart disease (7). The strict glycemic control, however, increases the incidence of severe hypoglycemia (8, 9) that increases the risk of cardiovascular diseases (10). On the other hand, a sustained hyperglycemia in the process of disease progression further impairs β-cell function (glucotoxicity) (11). Thus, diabetes treatment is required to maintain excellent glycemic control for a long time while avoiding the risk of hypoglycemia.

In recent years, new antidiabetic drugs, Dipeptidyl peptidase‐4 inhibitors (DPP4is) and Sodium-glucose cotransporter 2 inhibitors (SGLT2is), have been introduced. DPP-4is are safer than conventional insulin secretagogues with less risk of hypoglycemia (12). Similarly, since the hypoglycemic effect of SGLT2is is insulin-independent, they have a low risk of hypoglycemia and are effective in patients with high insulin resistance (13). In addition, it has been reported that DPP-4is and SGLT2is possess various beneficial effects other than improving glycemic control (14). Regarding pancreatic β-cell function, a recent meta-analysis reported that DPP-4is improve Homeostasis Model Assessment (HOMA)-β, an indicator of β-cell function (15). Similarly, SGLT2is ipragliflozin (16) and dapagliflozin (17) improved disposition index (DI) during 75 g oral glucose tolerance test (OGTT), suggesting that these SGLT2is improve β-cell function. However, based on the results of basic studies, there are differences in the mechanisms by which DPP-4is (18) or SGLT2is (19) improve β-cell function, and it remains unclear how these differences affect β-cell function. In this study, we compared the effects of a 24-week treatment with the SGLT2i luseogliflozin and the DPP-4i teneligliptin on β-cell function in obese patients with type 2 diabetes who had not achieved their glycemic control goals.

## 2 Methods

### 2.1 Study design, approval, and ethics

This was an investigator-initiated, multicenter, active-controlled, open-label, randomized clinical trial conducted at Kawasaki Medical School Hospital (Kurashiki, Japan), Kawasaki Medical School General Medical Center (Okayama, Japan), and Iwamoto Medical Clinic (Zentsuji, Japan). The protocol (No. 19001) was approved by the Kawasaki Medical School Clinical Research Review Board (Certification No.: CRB6200004). The study (jRCTs061190008) was registered with the Japan Registry of Clinical Trials (jRCT; https://jrct.niph.go.jp), a Japanese clinical research database, and was conducted in accordance with the principles of the Declaration of Helsinki (7th revision, 2013). The data management tasks in this study were performed by Yuka Nogami, a research assistant affiliated with Division of Diabetes, Endocrinology and Metabolism, Kawasaki Medical School. To ensure the quality of this study, monitoring and auditing were conducted by a third-party organization, Soiken Inc (Osaka, Japan).

### 2.2 Participants

The study period was from April 1, 2019 to December 31, 2022, and the case enrollment period was from April 1, 2019 to October 31, 2020. Inclusion criteria for subjects are as follows. 1) Japanese subjects who are at least 20 years of age and less than 80 years of age at acquisition of consent 2) patients with type 2 diabetes with HbA1c ≥ 7.0% and < 9.0% despite at least 12 weeks of treatment with diet and exercise (drug naïve) or a hypoglycemic agent other than a DPP-4 inhibitors or SGLT2 inhibitors 3) patients who have not started a new diabetic agent or changed (increased) the dose of any anti-diabetic agents for at least 12 weeks prior to obtaining consent 4) patients with a body mass index (BMI) of 20 kg/m^2^ or greater. While patients not taking insulin secretagogues (sulfonylureas and glinides) were preferred as much as possible, patients using sulfonylureas were included if they were using daily doses of up to 2 mg of glimepiride, 40 mg of gliclazide, and 1.25 mg of glibenclamide. In cases with a history of prior prescriptions for DPP-4 inhibitors and SGLT2 inhibitors, they were allowed entry if they had not used these drugs for at least 12 weeks prior to the acquisition of consent. Exclusion criteria for subjects are as follows. 1) patients with type 1 diabetes 2) patients with diabetes caused by a specific mechanism or disease (pancreatic exocrine disease, endocrine disease, drug-induced disorder, hereditary disorder) 3) patients with diabetic ketoacidosis, hyperosmotic hyperglycemia syndrome 4) patients who were using any insulin preparation or GLP-1 analogue 5) patients with myocardial infarction or stroke within 12 weeks 6) patients with severe infections, before and after surgery, or severe trauma 7) patients with severe hepatic damage (AST or ALT > 5 times the upper limit of the reference value at the center) 8) patients with severe renal dysfunction (eGFR <30 mL/min/1.73 m^2^) 9) patients with dehydration (patients with complaints of dehydration) 10) patients with urinary tract or genital tract infections 11) Patients who are pregnant, breast-feeding, may be pregnant, or are planning to become pregnant 12) patients with a history of hypersensitivity or contraindications to the study drug or control drug 13) patients with malignant tumors or history of malignant tumors 14) patients deemed inappropriate by the physician. All participants provided written informed consent to participate in this study.

### 2.3 Randomization and masking

**Figure 1:**
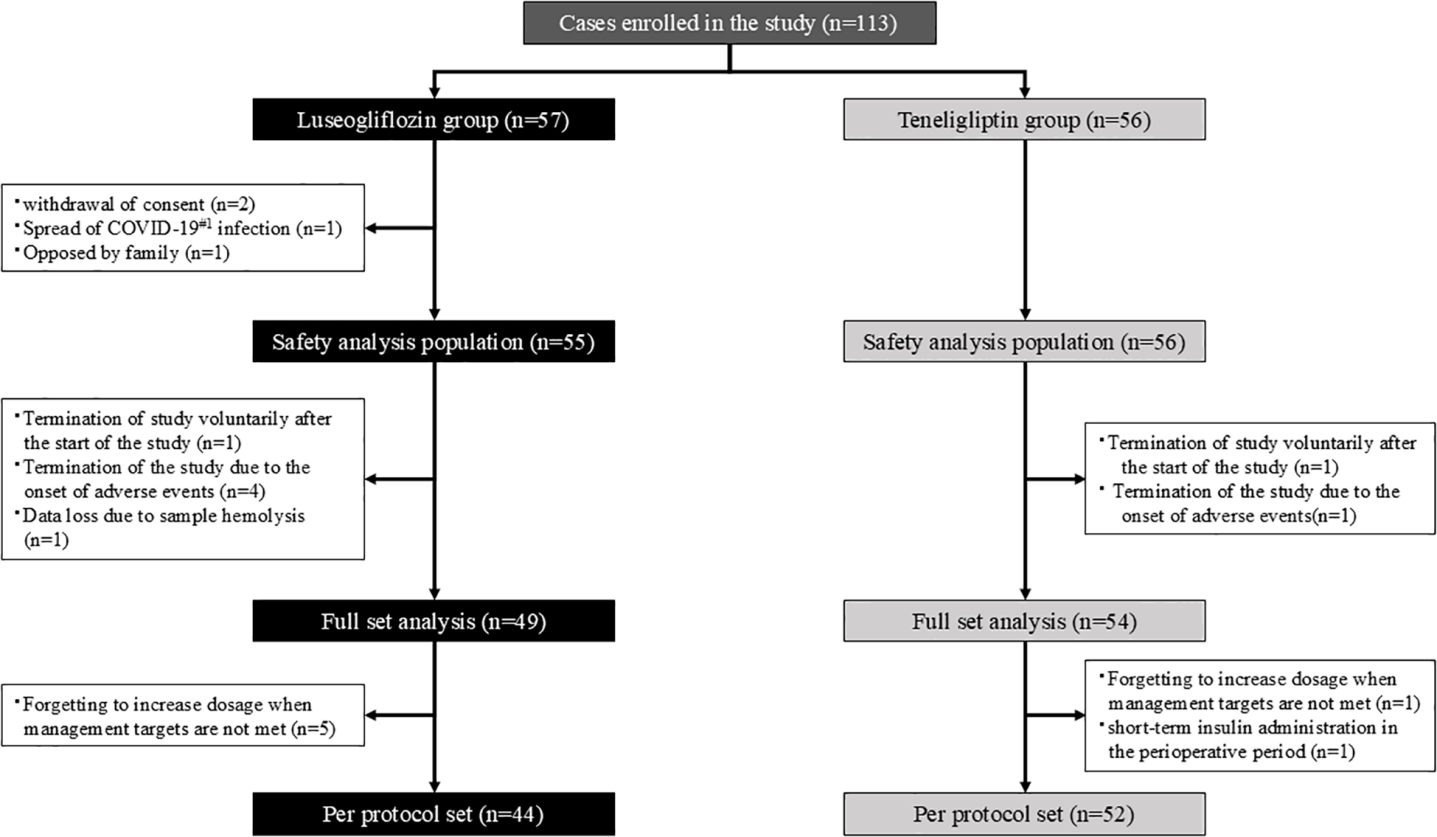
Participant flow. The white square notes the reason for the exclusion. #1. COVID-19; Coronavirus Disease 2019.

The flow of participants is shown in **Figure 1**. Participants were randomly assigned in a 1:1 ratio to the luseogliflozin or teneligliptin group using a computer-generated two-block randomization scheme with allocation factors such as age, BMI, and HbA1c level. The allocation task in this study was performed by Mrs. Yoshiko Oka, a research assistant at Division of Diabetes, Endocrinology and Metabolism, Kawasaki Medical School, who was not directly involved in the study.

### 2.4 Study procedures

**Figure 2:**
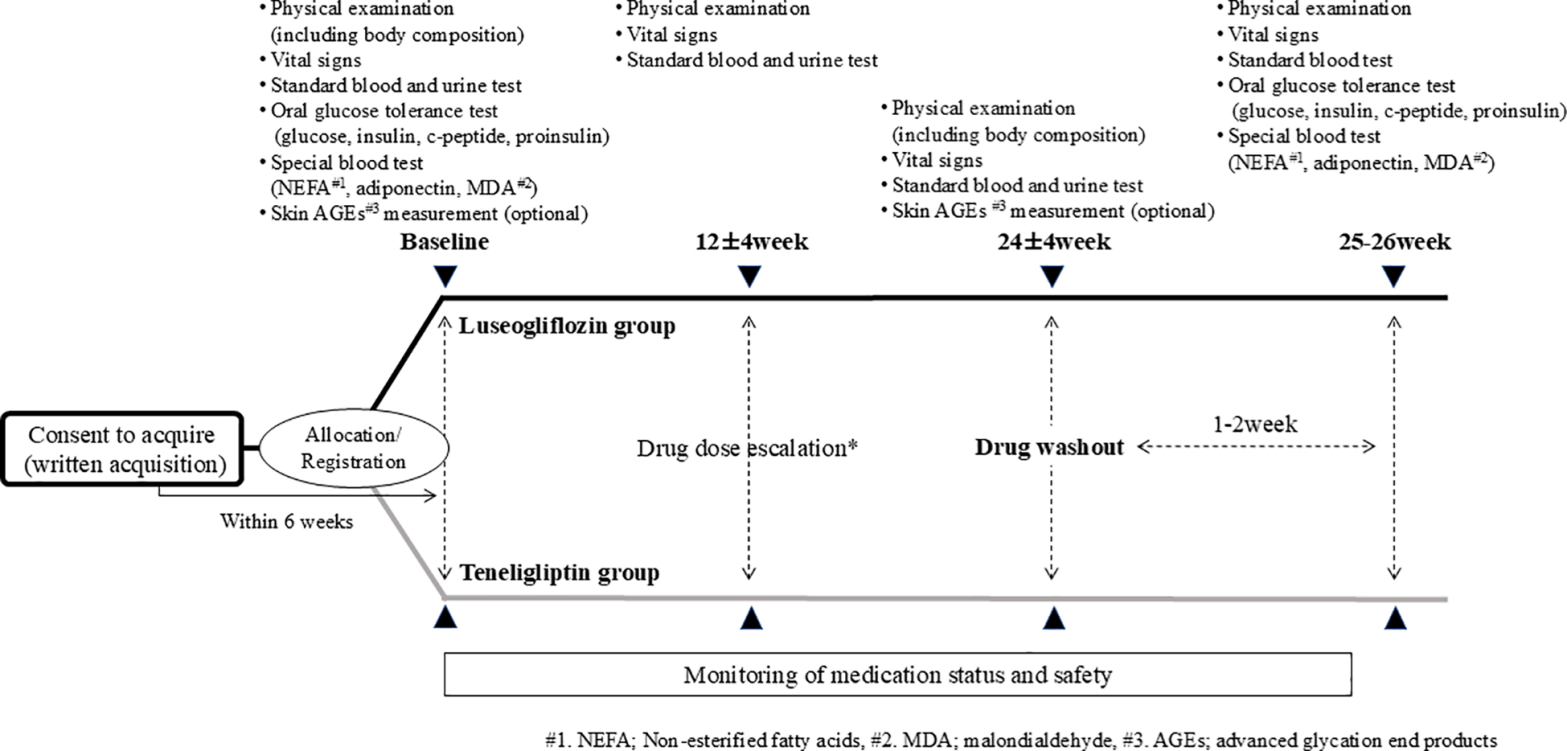
Study procedure. * If the HbA1c level is 7% or higher after 12 weeks of intervention, the drug dosage is increased by the attending physician’s decision, paying attention to side effects in accordance with the package insert.

The study procedure is shown in **Figure 2**. Participants visited the hospital within 6 weeks after consent and underwent various tests, including OGTT, prior to administration of the study drug. The study drugs were started orally at 2.5 mg once daily for luseogliflozin or 20 mg once daily for teneligliptin. The intervention period was 24 ± 4 weeks, and if the HbA1c level was not less than 7% after 12 weeks, the dose was increased to 5 mg or 40 mg, respectively. Participants were also tested including OGTT after the 24-week intervention but was washed out of each drug for 1-2 weeks after the intervention to eliminate the effects of the study drug as much as possible. In principle, any changes were not made to the drugs used other than the study drug during the study period. The study drug was discontinued when consent was withdrawn or when the principal investigator or a sub-investigator determined that discontinuation of the study was appropriate. Even in the case of discontinuation, observation was continued for safety evaluation as much as possible.

### 2.5 Outcomes

The primary endpoint is the change in DI (insulin) _0-120_ min from baseline to the 1-2 week washout point after the end of the intervention. DI is calculated as the product of insulin secretory capacity and insulin sensitivity based on the results of OGTT (20). DI is closely related to glucose tolerance and is useful as an indicator of insulin compensatory capacity against insulin resistance (20). DI (insulin) _0-120min._ was calculated by the following formula: DI (insulin) _0-120min._ = ((insulin _120min._ – insulin _0min._)/(glucose _120min._ - glucose _0min._)) × (Matsuda index). Matsuda index, an index of insulin resistance, was calculated by the following formula. Matsuda index= 10,000/square root of (fasting glucose × fasting insulin) × (mean glucose × mean insulin during OGTT) (21). The Key secondary endpoints were the change in DI (C-peptide) _0-120min.,_ DI (insulin) _0-30_ min., DI (C-peptide) _0-30_ min., serum proinsulin/C-peptide ratio, serum proinsulin/insulin ratio, and serum proinsulin concentration (Mercodia, Sweden), and the formulas are shown in **Supplementary Table 1**. Other evaluation parameters are listed in **Supplementary Table 2**.

### 2.6 Sample size calculation

Sample size was calculated as follows. According to a previous study that investigated the DI before and after treatment with SGLT2i, DI (insulin) _0-120min._ value of -0.91 ± 0.59 before treatment improved to -0.43 ± 0.48 after a 4-week intervention and a 1-week drug washout period (each value was log-transformed values) (16). On the other hand, DPP-4is have been reported to improve β-cell function indices immediately after the end of the intervention, but the improvement disappeared after a washout period (22, 23). Therefore, we assumed that the change in log-transformed DI (insulin) _0-120min._ after a 1- to 2-week washout of the study drug would be 0.48 ± 0.76 in the SGLT2i group and 0.05 ± 0.76 in the DPP-4i group, which represents an expected 5% improvement in DI before log transformation. Under a significance level (two-tailed test) of 5% and a power of 80%, there were 51 cases in one group and 102 cases in two groups to detect a significant difference between the two groups. Furthermore, assuming a dropout rate of 10%, we set enrollment targets for this study with 57 cases in one group and 114 cases in two groups.

### 2.7 Statistical analysis

Analyses of the primary and secondary endpoints were performed using data from the full analysis set (FAS), which included all study participants who were enrolled in the study and randomly assigned to study treatment. Participants were excluded if they received treatment with severe protocol violations, withdrew consent at any time, or had no data for the primary endpoint. Sensitivity analysis of the primary efficacy endpoint was performed using data from the per-protocol set (PPS), which excluded data from patients who discontinued the study intervention. The safety analysis set consisted of study subjects who were enrolled in the study, started treatment as assigned, and received part or all of the study treatment.

The primary efficacy endpoint was evaluated for statistical significance of group differences using FAS. We used analysis of covariance with the groups as fixed effects and the baseline value of DI and the allocation adjustment factors (age, HbA1c, and BMI) as covariates to test the null hypothesis that the change in DI at washout between the two groups was equal. A Student’s t-test was also performed as a sensitivity analysis. In addition, summary statistics of the change in DI after washout were calculated for each group, and one-sample t-test test was conducted for the significance of the change within each group. The analysis methods for secondary endpoints and exploratory outcomes are described in the Supporting information section. Comparison of the frequency of adverse events between treatment groups was performed using the Fisher’s direct probability test method. We analyzed continuous variables using log-transformed values as needed. All *p*-values were tested with a two-tailed test and were considered statistically significant when the *p*-value was less than 0.05. To ensure the reliability of the analysis results, statistical analyses were performed by a third-party organization, Soiken Inc. (Osaka, Japan). All statistical analyses were performed with SAS version 9.4.

## 3 Results

### 3.1 Analysis population

As shown in **Table 1**, there were no statistically significant differences in patient characteristics between the randomly assigned luseogliflozin and teneligliptin groups. Briefly describing the clinical background of each group, each group was in their early 60s, had diabetes for approximately 10 years, body mass index of approximately 27 kg/m^2^, HbA1c level of approximately 7.5% (59.0 mmol/mol), and no high incidence of microvascular or macrovascular complications. The number of categories of glucose-lowering drugs which have been administered are as follows: 0.9 ± 0.8 in luseogliflozin group and 1.0 ± 0.9 in teneligliptin group, with no statistical difference between the two groups. Drug naïve cases in luseogliflozin and teneligliptin group were 33% and 35%, respectively, and the main glucose-lowering drugs previously administered were biguanides in both groups. There were no statistical differences in the prevalence of other comorbidities or the use of therapeutic agents. The Matsuda index, C-peptide index and DI were not significantly different between the two groups.

**Table 1:** Baseline characteristics in this study subjects.

	Luseogliflozin (n=49)	Teneligliptin (n=54)	*p*-value
Age (year)	60.8 ± 11.1	62.6 ± 11.2	0.41
Gender (male)	26 (53.1)	31 (57.4)	0.70
Diabetes duration (year)	10.1 ± 7.9	9.2 ± 7.6	0.57
Height (cm)	161.1 ± 9.3	161.7 ± 9.0	0.74
Body weight (kg)	69.9 ± 12.3	71.9 ± 19.1	0.54
Body mass index (kg/m^2^)	27.0 ± 4.2	27.2 ± 5.4	0.78
Skeletal muscle mass (kg)	24.8 ± 4.5	25.5 ± 6.5	0.57
Visceral fat area (cm^2^)	122.3 ± 47.5	120.8 ± 57.4	0.89
HbA1c (%)	7.6 ± 0.4	7.5 ± 0.5	0.50
HbA1c (mmol/mol)	59.3 ± 4.9	58.6 ± 5.4	0.50
Fasting plasma glucose (mg/dL)	149.3 ± 23.4	147.2 ± 17.3	0.60
Serum insulin (μIU/mL)	9.2 ± 8.3	7.8 ± 5.1	
Ln serum insulin (μIU/mL)	1.88 ± 0.85	1.83 ± 0.69	0.75
Serum C-peptide (ng/mL)	2.06 ± 0.97	2.09 ± 0.92	
Ln serum C-peptide (ng/mL)	0.63 ± 0.43	0.65 ± 0.42	0.81
C-peptide index	1.37 ± 0.56	1.41 ± 0.61	
Ln C-peptide index	0.24 ± 0.40	0.26 ± 0.41	0.76
Disposition index	0.80 ± 0.60	0.92 ± 0.59	
Ln disposition index	-0.46 ± 0.68	-0.26 ± 0.60	0.12
Matsuda index	5.53 ± 4.87	5.08 ± 2.95	
Ln Matsuda index	1.42 ± 0.78	1.45 ± 0.60	0.81
Total cholesterol (mg/dL)	183.0 ± 37.5	191.4 ± 30.9	0.22
Triglyceride (mg/dL)	129.3 ± 67.0	119.7 ± 60.0	
Ln Triglyceride (mg/dL)	4.74 ± 0.49	4.67 ± 0.48	0.46
HDL-cholesterol (mg/dL)	52.0 ± 12.0	56.0 ± 12.3	0.09
LDL- cholesterol (mg/dL)	105.2 ± 30.2	111.4 ± 28.9	0.29
SBP (mmHg)	130.5 ± 14.0	133.6 ± 14.6	0.30
DBP (mmHg)	77.7 ± 9.7	78.9 ± 12.9	0.62
Vascular complications			
Ischemic heart disease	2 (4.1)	2 (3.7)	1.00
Cerebrovascular disease	1 (2.0)	3 (5.6)	0.62
Peripheral arterial disease	0 (0.0)	1 (1.9)	1.00
Neuropathy	7 (18.4)	3 (7.1)	0.18
Retinopathy	6 (14.6)	5 (10.2)	0.54
None	35 (85.4)	44 (89.8)	0.80
Simple	3 (7.3)	2 (4.1)	
Pre-proliferative	1 (2.4)	2 (4.1)	
Proliferative	2 (4.9)	1 (2.0)	
Nephropathy ≥Stage 2	13 (26.5)	19 (35.2)	0.40
Stage 1	36 (73.5)	35 (64.8)	0.52
Stage 2	10 (20.4)	16 (29.6)	
Stage 3	3 (6.1)	3 (5.6)	
≥ Stage 4	0 (0.0)	0 (0.0)	
Comorbidity			
Dyslipidemia	35 (71.4)	37 (68.5)	0.83
Hypertension	31 (63.3)	30 (55.6)	0.55
NAFLD	6 (12.2)	10 (18.5)	0.43
ALD	2 (4.1)	3 (5.6)	1.00
Glucose-lowering agents			
None	16 (32.7)	19 (35.2)	0.84
Sulfonylurea	4 (8.2)	5 (9.3)	1.00
Glinide	4 (8.2)	5 (9.3)	1.00
Biguanide	30 (61.2)	30 (55.6)	0.69
Thiazolidine	10 (20.4)	5 (9.3)	0.16
α-glucosidase inhibitor	2 (4.1)	3 (5.6)	1.00
Anti-hypertensive agents			
None	24 (49.0)	27 (50.0)	1.00
ARB	23 (46.9)	22 (40.7)	0.56
ACE inhibitor	0 (0.0)	0 (0.0)	–
CCB	21 (42.9)	18 (33.3)	0.42
Diuretic	0 (0.0)	0 (0.0)	–
β-blocker	2 (4.1)	2 (3.7)	1.00
α-blocker	0 (0.0)	0 (0.0)	–
Others	0 (0.0)	1 (1.9)	1.00
Lipid-lowering agents			
None	16 (32.7)	21 (38.9)	0.54
Statin	32 (65.3)	28 (51.9)	0.23
Fibrate	4 (8.2)	3 (5.6)	0.71
SPPARMα	0 (0.0)	2 (3.7)	0.50
Ezetimib	2 (4.1)	3 (5.6)	1.00
Probucol	0 (0.0)	0 (0.0)	–
Ion-exchange resin	0 (0.0)	0 (0.0)	–
Nicotinic acid derivative	0 (0.0)	1 (1.9)	1.00
PUFA	0 (0.0)	0 (0.0)	–
Other agents			
Antiplatelet drug	2 (4.1)	4 (7.4)	0.68
Anticoagulant	0 (0.0)	1 (1.9)	1.00

HDL; high-density lipoprotein, LDL; low-density lipoprotein, SBP; systolic blood pressure, DBP; diastolic blood pressure, NAFLD; nonalcoholic fatty liver disease, ALD; alcoholic liver disease, ARB; angiotensin II receptor blocker, ACE; angiotensin-converting enzyme, CCB; calcium channel blocker, SPPARMα; selective peroxisome proliferator-activated receptor α modulator, PUFA; polyunsaturated fatty acids, Ln; logarithmus naturalis.

### 3.2 Changes in insulin secretory capacity, insulin resistance, and glucose tolerance with drug intervention

**Figure 3:**
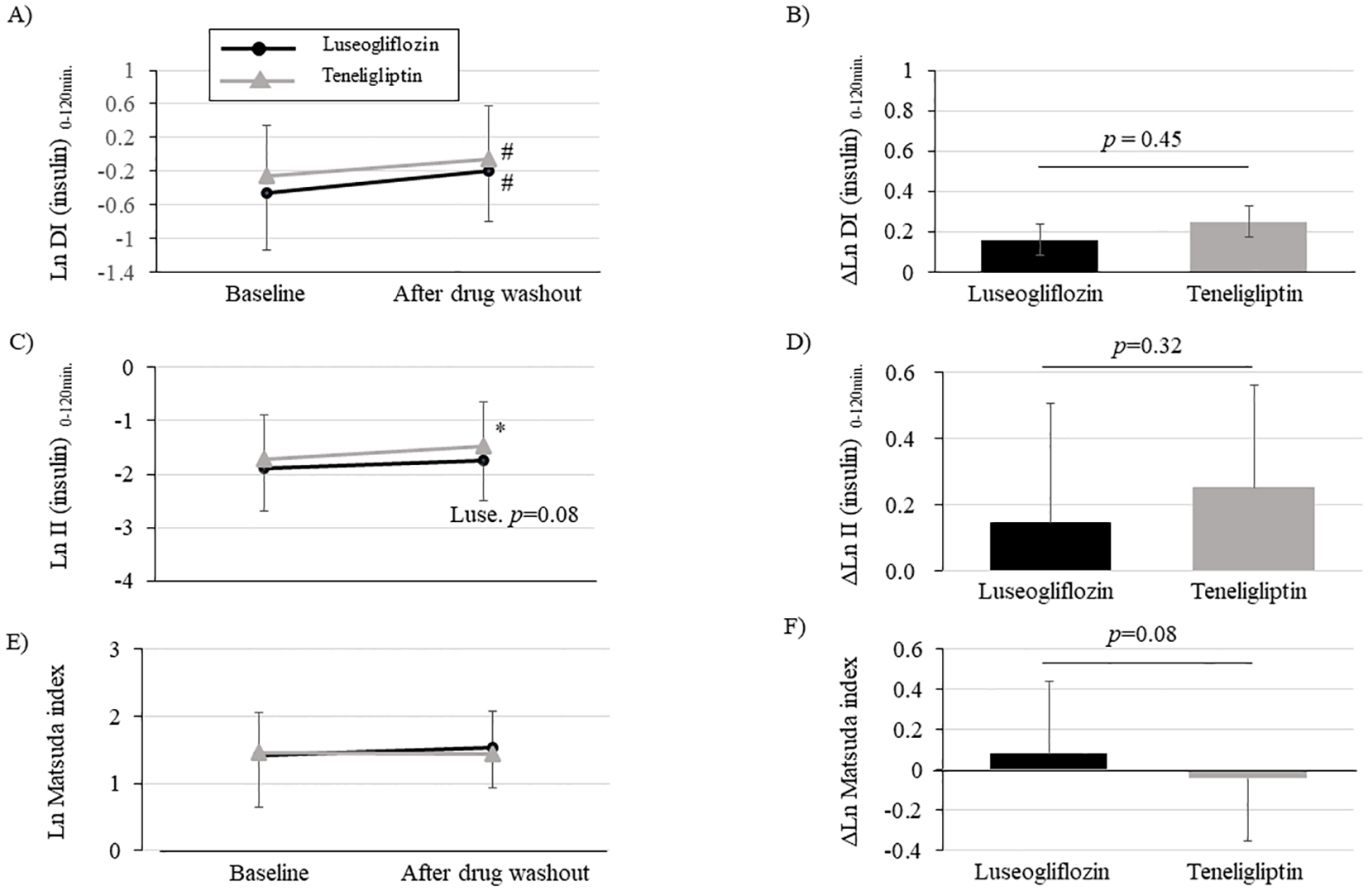
Changes in disposition index (DI), insulinogenic index (II) and Matsuda index after intervention with luseogliflozin or teneligliptin. Each figure **(A-F)** indicates DI (insulin) _0-120min._ **(A, B)**, II (insulin) _0-120min._ **(C, D)**, Matsuda index **(E, F)**. Changes over time from baseline **(A, C, E)** and the amount of change (**B, D, F**; post-washout values minus values at baseline) are shown. Parameters that were non-normally distributed are shown as natural logarithms. # *p*<0.05 vs. baseline, *p<0.001 vs. baseline.

We performed an OGTT with a washout of the study drug after the intervention to eliminate the effects of the study drug on insulin secretion capacity, insulin resistance, and glucose tolerance. The drug interventions with luseogliflozin or teneligliptin significantly improved DI (insulin) _0-120_ min., assessed using insulin measurements, compared to pre-intervention (**Figure 3A**; **Supplementary Table 3**). There were no statistically significant group differences in the amount of change (**Figure 3B**; **Supplementary Table 3**). We performed sensitivity analysis (**Supplementary Table 3**) using PPS for the change in DI (insulin) _0-120_ min. and found that the results were consistent with the analysis using FAS. On the other hand, DI (C-peptide) _0-120_ min., evaluated using C-peptide measurements, was significantly improved only in luseogliflozin group, although there was no difference in the amount of change between the groups (**Supplementary Table 3**). The (insulin) DI _0-30min._ was significantly improved only in teneligliptin group (**Supplementary Table 3**), while DI (C-peptide) _0-30min._ was significantly improved in both groups (**Supplementary Table 3**).

Evaluation of the components of DI (insulin) _0-120min._ calculation showed that insulinogenic index (insulin) _0-120min._ was significantly increased in teneligliptin group and tended to be increased in luseogliflozin group (**Figure 3C**; **Supplementary Table 3**). The insulinogenic index (C-peptide) _0-120_ min. was significantly improved in both groups (**Supplementary Table 3**). On the other hand, the Matsuda index tended to be improved only in luseogliflozin group (**Figure 3E**; **Supplementary Table 3**). Consistent with the results, adiponectin levels were significantly increased only in luseogliflozin group (**Supplementary Table 3**). However, there were no differences in changes in insulinogenic index or Matsuda index between the groups (**Figures 3D, F**; **Supplementary Table 3**).

**Figure 4:**
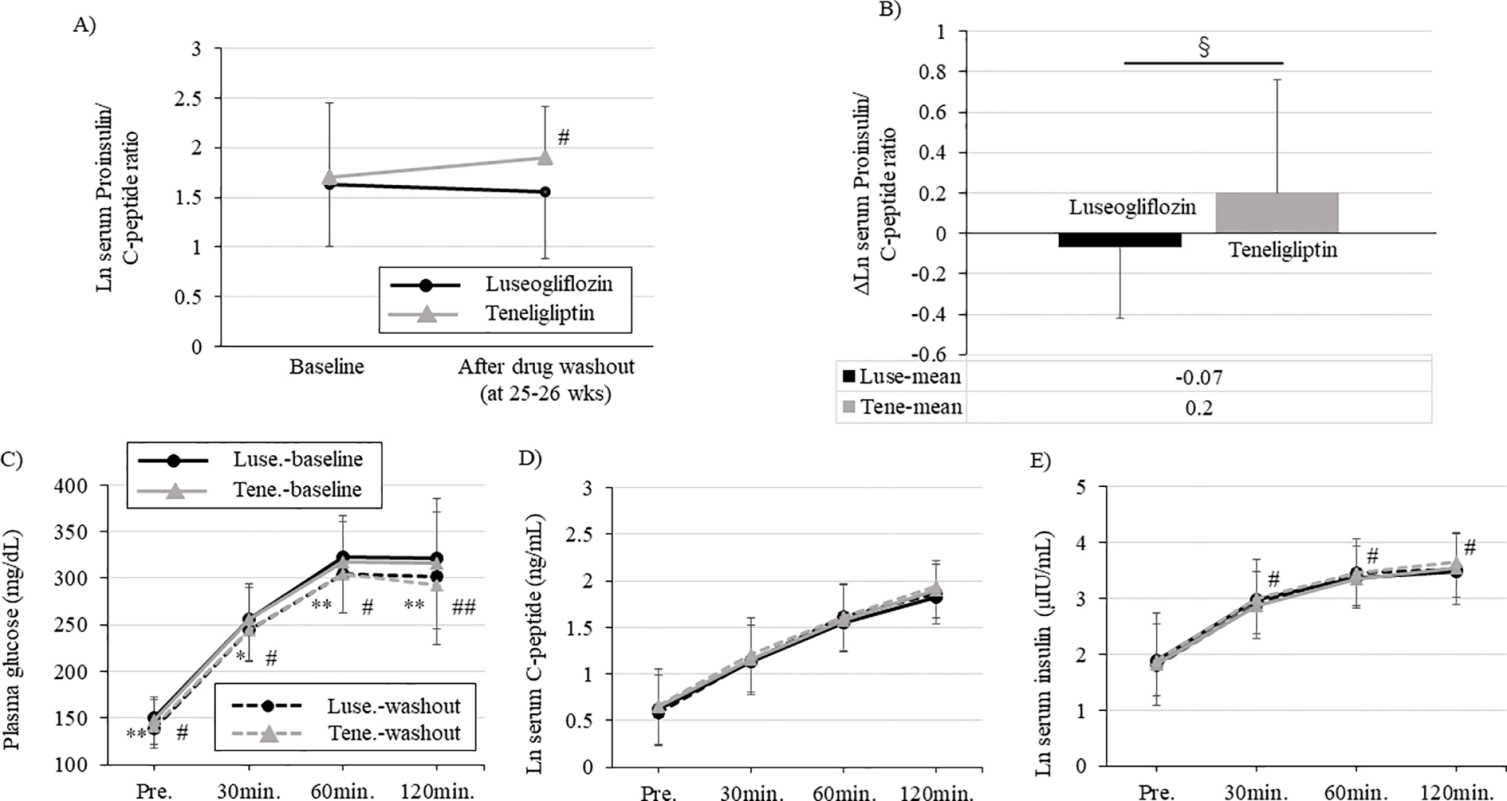
Changes in each parameter during oral glucose tolerance test and serum proinsulin/C-peptide ratio performed at baseline and after drug washout. Each figure **(A-C)** shows blood glucose **(A)**, serum C-peptide **(B)**, and serum insulin **(C)** levels during the oral glucose tolerance test. Solid black circle; luseogliflozin group at baseline, solid gray line; teneligliptin group at baseline, dotted black circle; luseogliflozin group after washout, dotted gray line; teneligliptin group after washout **(A, B, C)**. **(D)** Change in serum proinsulin/C-peptide ratio in each group. Solid black circle; luseogliflozin group, solid gray line; teneligliptin group. **(E)** Amount of change in serum proinsulin/C-peptide ratio in each group. Black bars; luseogliflozin group, gray bars; teneligliptin group. Parameters that were non-normally distributed are shown as natural logarithms. * *p*<0.05, ** *p*<0.005: baseline vs. after washout in luseogliflozin group, # *p*<0.05, ## *p*<0.005: baseline vs. after washout in teneligliptin group. § *p*<0.005: luseogliflozin vs. teneligliptin.

The proinsulin/C-peptide ratio, a biomarker of β-cell dysfunction, was significantly worse in teneligliptin group, while it tended to decrease in luseogliflozin group (**Figure 4A**; **Supplementary Table 3**). The change in proinsulin/C-peptide from the baseline was statistically significant between the two drugs and was improved with luseogliflozin. (**Figure 4B**; **Supplementary Table 3**). A similar trend was observed for changes in serum proinsulin concentration or serum proinsulin/insulin ratio (**Supplementary Table 3**).

Glycemic trends during the OGTT after drug washout were significantly improved in both groups compared to pre-intervention, with no differences between the groups (**Figure 4C**). The change in serum C-peptide concentration during the OGTT after drug washout was unchanged in both groups compared to baseline, and there was no difference between the two groups (**Figure 4D**). Maintaining serum C-peptide concentrations despite improved blood glucose levels in both groups suggested that glucose-responsive insulin secretion was improved. The change in serum insulin concentration during the OGTT after drug washout was significantly increased in teneligliptin group and maintained in luseogliflozin group compared to baseline (**Figure 4E**).

### 3.3 Changes in clinical parameters during the intervention period

**Figure 5:**
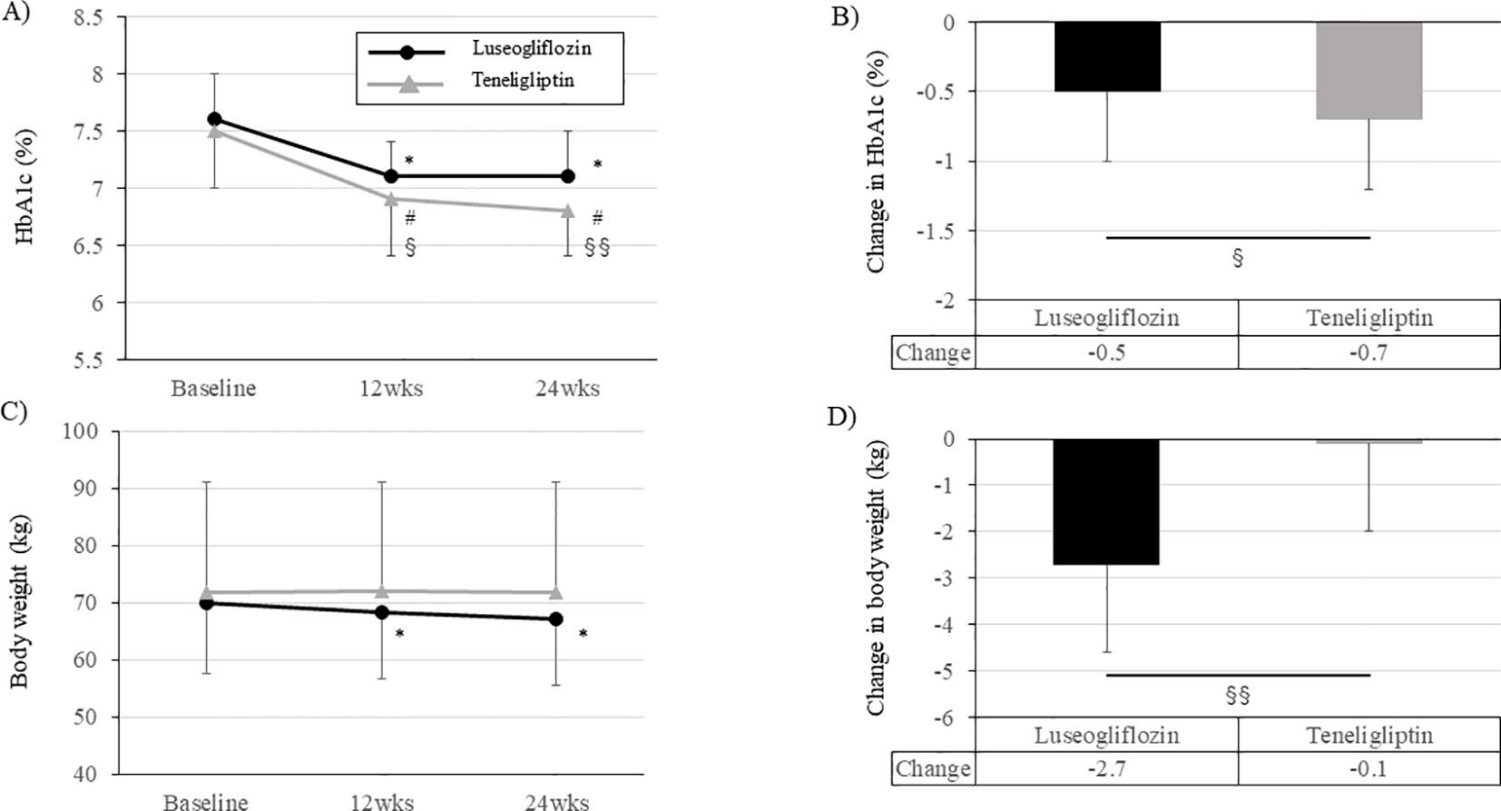
Change in HbA1c levels and body weight during the intervention period in each group. **(A, C)** Change in HbA1c levels **(A)** and body weight **(C)** in each group. Solid black circle; luseogliflozin group, solid gray line; teneligliptin group. **(B, D)** Amount of change in HbA1c levels **(B)** and body weight **(D)** in each group. Black bars; luseogliflozin group, gray bars; teneligliptin group. * p<0.001 vs. baseline in luseogliflozin group, # p<0.001 vs. baseline in teneligliptin group, § p<0.05, §§ p<0.001 luseogliflozin. vs. teneligliptin.

During the 24-week intervention period, both groups had a significant improvement in HbA1c level already at week 12 compared to baseline, and this effect was maintained up to 24 weeks (**Figure 5A**; **Supplementary Table 3**). However, the change in HbA1c during the intervention period was greater in teneligliptin group than in luseogliflozin group (**Figure 5B**; **Supplementary Table 3**). Fasting blood glucose level was improved significantly in both groups but were much lower in luseogliflozin group (**Supplementary Table 3**). The levels of total ketone bodies were significantly increased only in luseogliflozin group (**Supplementary Table 3**). Total ketone body level after drug washout in luseogliflozin group tended to be lower than that at baseline, supporting that the drug was adequately washed out. Body weight was not changed in teneligliptin group but was significantly reduced in luseogliflozin group (**Figures 5C, D**; **Supplementary Table 3**). Consistent with this result, the skeletal muscle mass or visceral fat area measured by anthropometry were significantly reduced, and serum adiponectin concentration was significantly increased only in the luseogliflozin group (**Supplementary Table 3**). Serum malondialdehyde concentration and skin Advanced glycation end products (AGEs) levels did not change significantly before and after the intervention in both groups (**Supplementary Table 3**).

### 3.4 Differences between drug groups in clinical background related to improvement of DI

We examine whether clinical parameters affect changes in DI in response to two drugs. As shown in **Supplementary Figure 1** and **2**, we divided the clinical background parameters mainly by median values and tested the impact of both drugs on the DI (insulin) _0-120min_. Result did not show significant difference between the drugs were identified in the clinical characteristics that are likely to improve DI in the present study. On the other hand, an improvement in DI was observed for both drugs in patients with younger, higher values of BMI, C-peptide index, and HbA1c, and lower values in DI. In addition, DI was improved in cases with more improved HbA1c, BMI, and proinsulin/C-peptide ratio values, i.e., those with the more alleviated β-cell burden.

### 3.5 Comparison in adverse events identified during the intervention period

As shown in **Supplementary Table 4**, adverse events were identified in 31 of 111 cases during the intervention period, with a significantly higher rate in luseogliflozin group. There were no cases of death identified in either group. There were two serious adverse events in luseogliflozin group and one in teneligliptin group, but they were not causally related to the study drug. Adverse events experienced by more than 3% of patients were genital itching, polyuria, cystitis, fatigue, dizziness, cavity, and upper respiratory tract infection in luseogliflozin group, and were upper respiratory tract infection and coughing in teneligliptin group.

## 4 Discussions

One of the unique features of this study was the examination of changes from baseline in DI under washout conditions of the study drug, i.e., changes in the compensatory insulin secretory capacity of net β-cells. Another unique feature is that this is the first clinical trial to directly compare the effects of SGLT2is and DPP-4is on net β-cell function.

The present study showed that both drugs had comparable effects on the DI under drug washout conditions after 24 weeks of treatment. This result probably indicates that the alleviation of glucotoxicity resulted in equivalent changes in the compensatory insulin secretion process during the process from insulin biosynthesis to secretion in β-cells. On the other hand, proinsulin was increased with teneligliptin but not with luseogliflozin, suggesting that luseogliflozin may have relieved the burden on β-cells during the insulin biosynthesis process. Then, when the insulinogenic index and the Matsuda index, which are necessary to calculate the DI, were analyzed separately, there were differences in the mechanisms by which both drugs improved compensatory insulin secretion. Teneligliptin improved insulin secretion without changing insulin sensitivity, whereas luseogliflozin improved insulin secretion while improving insulin sensitivity. The differences, as mentioned above, in serum proinsulin/C-peptide ratios between drugs may be related to the different mechanisms by which each drug improved DI. Although further investigation is needed, these results suggest that luseogliflozin may be more potent than teneligliptin in improving β-cell function when both drugs are used for extended periods.

Some basic studies have reported that DPP-4is protect β-cell mass and function via metabolic improvement and incretin (24, 25). On the other hand, previous clinical studies reported that DPP-4is required several years to improve the DI after drug washout (22, 23, 26). In the current study, teneligliptin improved the DI after drug washout, despite short-term intervention over a 6-month period. One of the factors contributing to this result may be related to the degree of impaired glucose tolerance at the start of the intervention. Subjects in previous clinical trials (22, 23, 26) were patients with very mild glucose intolerance, such as patients with pre-diabetes or mild type 2 diabetes. On the other hand, we recruited patients with slightly severe glucose intolerance than those in previous studies (22, 23, 26). We think that the present results are largely influenced by the improvement in glucose tolerance. Another factor responsible for the current result may be attributed to the length of the washout period. In previous reports, the washout period of study drug was 4 to 12 weeks in studies for patients with type 2 diabetes (23, 26) and 2 weeks in studies for patients with prediabetes (22). The drug washout period in our clinical study was approximately 2 weeks, which may have resulted in a smaller rebound in blood glucose levels due to a shorter washout period. We think that the present and previous clinical studies suggest that DPP-4is improve the net function of β-cells in patients with type 2 diabetes, and that the effect is greater with longer durations of treatment.

Several basic studies have reported that DPP-4is reduce β-cell injury by reducing oxidative stress (18, 27) and endoplasmic reticulum stress (18, 24, 25). Furthermore, meta-analysis that tested the efficacy of DPP-4is versus placebo reported that DPP-4is improved the proinsulin/insulin ratio during drug treatment (28). Although our study was not placebo-controlled trial, the lack of improvement in proinsulin/C-peptide ratio and proinsulin/insulin ratio after teneligliptin treatment may be due in part to the effect of increased blood glucose levels due to drug washout.

DPP-4is are insulin secretagogues and therefore may impose a certain load on β-cells. In contrast, SGLT2is reduce insulin demand on β-cells by improving insulin sensitivity through weight loss, in addition to promoting urinary glucose excretion (29). Basic studies using SGLT2 knockout mice (30) and SGLT2i-treated mice (19, 31–33) have demonstrated that SGLT2 inhibition protects β-cell mass and function by reducing metabolic stresses such as oxidative stress and endoplasmic reticulum stress. Several clinical trials, including single-arm (34) or placebo-controlled trials (17, 35), have also reported that SGLT2is improve β-cell glucose sensitivity. Takahara et al. also reported that ipragliflozin improves DI not only on treatment but also after 1 week of drug washout (16). In the present study, we evaluated the change in DI after a washout period of approximately 2 weeks and found that the luseogliflozin group showed only a trend toward improvement. The reason for just showing a trend was presumably due to the mild severity of glucose intolerance prior to the intervention and the longer duration of drug washout than that in previous studies (16).

On the other hand, in our study, luseogliflozin significantly reduced the proinsulin/C-peptide ratio even after washout, which is consistent with the results of a previous report that was performed without a defined washout period (36, 37). The previous report and our results suggest that SGLT2is have protective effects on β-cells. We initially designed the clinical trial with the hypothesis that luseogliflozin would improve DI more than teneligliptin, but the effects were comparable. The results were consistent with the results of sub-analysis from the phase 3 trial using canagliflozin (35), which was conducted without a defined washout period. However, the similar improvement in DI despite a small but significant improvement in HbA1c in teneligliptin group may be related to a significant improvement in the proinsulin/C-peptide ratio in luseogliflozin group.

Our study has several limitations. First, this study is an open-label design that includes two open-label active treatment groups without a placebo group. Therefore, we cannot rule out the possibility that the improvement in DI in both groups is attributable to improvements in lifestyle. Second, it is unclear what the results would be in a longer-term intervention because the intervention period was limited to 24 weeks. Third, although the current study defined a drug washout period, it is difficult to address the protective effect of the drug on β-cells in a glucose-independent manner because the subjects were individuals with type 2 diabetes who had not yet achieved their glycemic control goals. Finally, the study population is limited to Japanese patients with type 2 diabetes and may not be applicable to other ethnic populations.

## 5 Conclusions

Improvement of disposition index in subjects with obese type 2 diabetes was comparable between luseogliflozin and teneligliptin. On the other hand, it is likely that alleviation of β-cell dysfunction is more effective with luseogliflozin compared to tenegliptin.

## Data Availability

The data supporting the results of this study are not being disclosed due to confidentiality and are available from the corresponding author upon reasonable request.

## Supplementary material

Supplementary Figure 1 Differences in efficacy between the two drugs in the ln disposition index, grouped by the median of each clinical parameter at baseline. Black bars; luseogliflozin group, gray bars; teneligliptin group. One-sample t-tests were performed for significance of change from baseline within each category. Student’s t-tests were performed on the significance of the change between each category. * *p*<0.05, ** *p*<0.001: Intra-group comparison of luseogliflozin; # *p*<0.05, ## *p*<0.01: Intra-group comparison of teneligliptin. BMI, body mass index; DI, disposition index; Proins./Cpep, Proinsulin/C-peptide; NEFA, Non-esterified fatty acids; AGEs, advanced glycation end products; Ln, logarithmus naturalis.

Supplementary Figure 2 Difference in efficacy between the two drugs in the ln disposition index, grouped by the median change in each clinical parameter. Black bars; luseogliflozin group, gray bars; teneligliptin group. One-sample t-tests were performed for significance of change from baseline within each category. Student’s t-tests were performed on the significance of the change between each category. * *p*<0.05, ** *p*<0.001: Intra-group comparison of luseogliflozin; # *p*<0.05, ### *p*<0.005: Intra-group comparison of teneligliptin. BMI, body mass index; DI, disposition index; Proins./Cpep, Proinsulin/C-peptide; NEFA, Non-esterified fatty acids; Ln, logarithmus naturalis.
