# Supplementary figures and images for: Comparison of protective effects of teneligliptin and luseogliflozin on pancreatic β-cell function: randomized, parallel-group, multicenter, open-label study (SECRETE-I study)
Source: Front Endocrinol (Lausanne). 2024 Oct 21;15:1412553. doi: 10.3389/fendo.2024.1412553 (PMC11532122; doi:10.3389/fendo.2024.1412553)

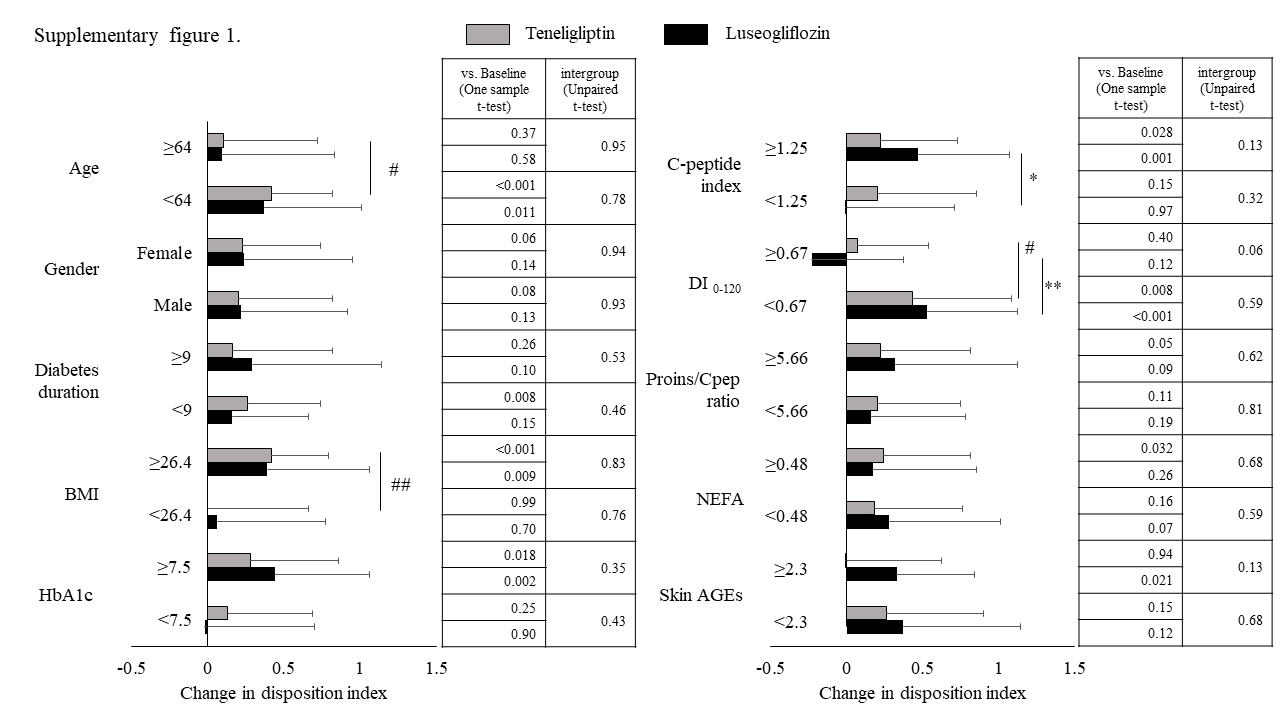

Supplement: Supplementary Figure 1 — Differences in efficacy between the two drugs in the ln disposition index, grouped by the median of each clinical parameter at baseline. Black bars; luseogliflozin group, gray bars; teneligliptin group. One-sample t-tests were performed for significance of change from baseline within each category. Student’s t-tests were performed on the significance of the change between each category. * p<0.05, ** p<0.001: Intra-group comparison of luseogliflozin; # p<0.05, ## p<0.01: Intra-group comparison of teneligliptin. BMI, body mass index; DI, disposition index; Proins./Cpep, Proinsulin/C-peptide; NEFA, Non-esterified fatty acids; AGEs, advanced glycation end products; Ln, logarithmus naturalis. [file Image1.tif]

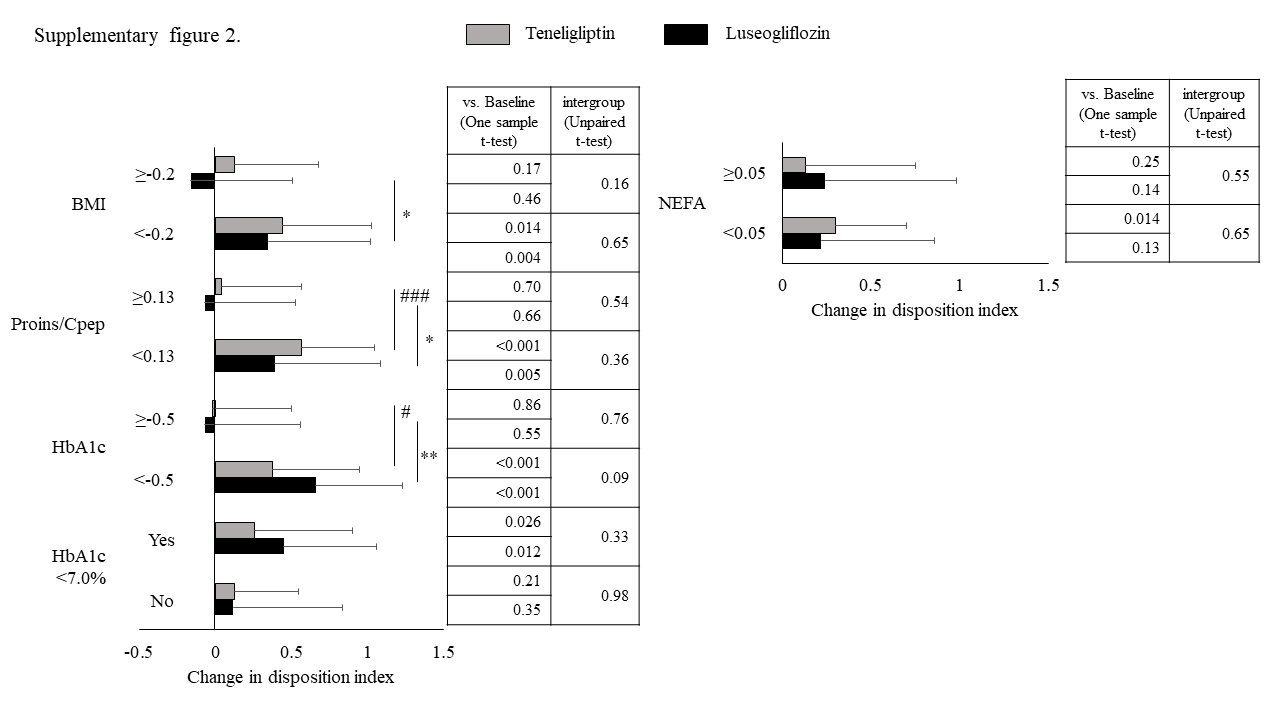

Supplement: Supplementary Figure 2 — Difference in efficacy between the two drugs in the ln disposition index, grouped by the median change in each clinical parameter. Black bars; luseogliflozin group, gray bars; teneligliptin group. One-sample t-tests were performed for significance of change from baseline within each category. Student’s t-tests were performed on the significance of the change between each category. * p<0.05, ** p<0.001: Intra-group comparison of luseogliflozin; # p<0.05, ### p<0.005: Intra-group comparison of teneligliptin. BMI, body mass index; DI, disposition index; Proins./Cpep, Proinsulin/C-peptide; NEFA, Non-esterified fatty acids; Ln, logarithmus naturalis. [file Image2.tif]
