# Supplementary material for: Comparison of protective effects of teneligliptin and luseogliflozin on pancreatic β-cell function: randomized, parallel-group, multicenter, open-label study (SECRETE-I study)
Source: Front Endocrinol (Lausanne). 2024 Oct 21;15:1412553. doi: 10.3389/fendo.2024.1412553 (PMC11532122; doi:10.3389/fendo.2024.1412553)
Supplement: Supplementary file 3 [file Table1.docx]

Supplementary table 1：Formula for calculating key secondary endpoints

| **Indicator** | **Calculating Formula** |
| --- | --- |
| DI (C-peptide) _0-120min._ | = [(C-peptide _120min._ – C-peptide _0min._) / (glucose _120min._ - glucose _0min._)] × (Matsuda index) |
| DI (insulin) _0-30 min._ | = [(insulin _30min._ – insulin _0min._) / (glucose _30min._ - glucose _0min._)] × (Matsuda index) |
| DI (C-peptide) _0-30 min._ | = [(C-peptide _30min._ – C-peptide) _0min._) / (glucose _30min._ - glucose _0min._)] × (Matsuda index) |
| Serum proinsulin/ serum C-peptide ratio | = Serum proinsulin _0min._ / serum C-peptide _0min._ |
| Serum proinsulin/ serum insulin ratio | Serum proinsulin _0min._ / serum insulin _0min._ |

The change in each indicator was calculated by subtracting the baseline value from the value after the drug washout. DI; disposition index
