# Supplementary material for: Comparison of protective effects of teneligliptin and luseogliflozin on pancreatic β-cell function: randomized, parallel-group, multicenter, open-label study (SECRETE-I study)
Source: Front Endocrinol (Lausanne). 2024 Oct 21;15:1412553. doi: 10.3389/fendo.2024.1412553 (PMC11532122; doi:10.3389/fendo.2024.1412553)
Supplement: Supplementary file 4 [file Table2.docx]

Supplementary Table 2. Other clinical parameters examined in this study

|  | Parameters |
| --- | --- |
| Blood test | = Glucose metabolism related parameters =  HbA1c, Blood glucose, Insulin, C-peptide (CPR), Proinsulin, Ketone bodies, Adiponectin (SRL, Japan) |
|  | = Other parameters =  Malondialdehyde (Cell Biolabs, Inc., United States) |
| Others | Body weight, BMI, Body composition (InBody770; Inbody Japan, Japan), Skin advanced glycation end products (AGEs; AGE Reader mu; Diagnoptics, Nederland) |
| Safety assessment^*1^ | |

^*1^ For the purposes of safety assessment, any unfavorable or unintended medical events were considered "adverse events". The severity was judged in accordance with the "Ethical Guidelines for Medical Research Involving Human Subjects” (December 22, 2014, Ministry of Education, Culture, Sports, Science and Technology/Ministry of Health, Labour and Welfare). The criteria for "serious" were as follows: 1) death, 2) life-threatening, 3) requiring hospitalization or prolonged hospitalization for treatment, 4) permanent or significant disability or dysfunction, and 5) resulting in congenital abnormalities in offspring.
