# Supplementary material for: Comparison of protective effects of teneligliptin and luseogliflozin on pancreatic β-cell function: randomized, parallel-group, multicenter, open-label study (SECRETE-I study)
Source: Front Endocrinol (Lausanne). 2024 Oct 21;15:1412553. doi: 10.3389/fendo.2024.1412553 (PMC11532122; doi:10.3389/fendo.2024.1412553)
Supplement: Supplementary file 5 [file Table3.docx]

Supplementary table 3. Changes in parameters before and after intervention with luseogliflozin or teneligliptin

|  | Luseogliflozin  (n=49) | | | Teneligliptin  (n=54) | | | Between-group  difference | *p*-value |
| --- | --- | --- | --- | --- | --- | --- | --- | --- |
|  | n | Mean±SD  (95%CI) | One-sample  t-test | n | Mean±SD  (95%CI) | One-sample  t-test | Mean  (95%CI) |  |
| Disposition index (insulin) _0-120min._ (Full analysis set) | | | | | | | | |
| Baseline | 48 | 0.80±0.60  (0.62, 0.97) |  | 52 | 0.92±0.59  (0.75, 1.08) |  |  |  |
| After drug  washout | 47 | 0.96±0.53  (0.81, 1.12) |  | 53 | 1.13±0.67  (0.94, 1.31) |  |  |  |
| Change | 46 | 0.14±0.61  (-0.04, 0.32) |  | 51 | 0.21±0.62  (0.04, 0.39) |  |  |  |
| Ln disposition index (insulin) _0-120min._ (Full analysis set) | | | | | | | | |
| Baseline | 48 | -0.46±0.68  (-0.66, -0.27) |  | 52 | -0.26±0.60  (-0.43, -0.10) |  | -0.20  ( -0.45, 0.06) | 0.12 |
| After drug  washout | 47 | -0.20±0.59  (-0.37, -0.02) |  | 53 | -0.05±0.62  (-0.22, 0.12) |  | -0.14  ( -0.38, 0.10) | 0.24 |
| Change | 46 | 0.23±0.70  (0.02, 0.44) | 0.03 | 51 | 0.21±0.57  (0.05, 0.37) | 0.01 | 0.02  ( -0.24, 0.27) | 0.90 |
| Disposition index (insulin) _0-120min._ (Per-protocol set) | | | | | | | | |
| Baseline | 43 | 0.79±0.60  (0.61, 0.98) |  | 50 | 0.92±0.59  (0.75, 1.09) |  |  |  |
| After drug  washout | 42 | 1.01±0.54  (0.84, 1.17) |  | 51 | 1.15±0.67  (0.96, 1.34) |  |  |  |
| Change | 41 | 0.18±0.58  (0.00, 0.37) |  | 49 | 0.24±0.61  (0.06, 0.42) |  |  |  |
| Ln disposition index (insulin) _0-120min._ (Per-protocol set) | | | | | | | | |
| Baseline | 43 | -0.46±0.66  (-0.66, -0.26) |  | 50 | -0.26±0.60  (-0.44, -0.09) |  | -0.19  (-0.45, 0.07) | 0.14 |
| After drug  washout | 42 | -0.15±0.59  (-0.34, 0.03) |  | 51 | -0.02±0.60  (-0.19, 0.15) |  | -0.13  (-0.38, 0.12) | 0.30 |
| Change | 41 | 0.26±0.62  (0.07, 0.46) | 0.01 | 49 | 0.24±0.56  (0.08, 0.41) | 0.003 | 0.02  (-0.23, 0.27) | 0.88 |
| Disposition index (CPR) _0-120min._ | | | | | | | | |
| Baseline | 48 | 0.15±0.14  (0.11, 0.20) |  | 52 | 0.16±0.11  (0.13, 0.19) |  |  |  |
| After drug  washout | 47 | 0.17±0.13  (0.14, 0.21) |  | 53 | 0.19±0.15  (0.14, 0.23) |  |  |  |
| Change | 46 | 0.01±0.10  (-0.01, 0.04) |  | 51 | 0.03±0.13  (-0.005, 0.07) |  |  |  |
| Ln disposition index (CPR) _0-120min._ | | | | | | | | |
| Baseline | 48 | -2.23±0.87  (-2.48, -1.98) |  | 52 | -2.05±0.63  (-2.23, -1.88) |  | 0.18  (-0.12, 0.48) | 0.24 |
| After drug  washout | 47 | -1.98±0.68  (-2.18, -1.78) |  | 53 | -1.92±0.67  (-2.11, -1.74) |  | 0.05  (-0.21, 0.32) | 0.69 |
| Change | 46 | 0.20±0.55  (0.04, 0.37) | 0.02 | 51 | 0.13±0.55  (-0.02, 0.29) | 0.09 | -0.07  (-0.29, 0.15) | 0.53 |
| Disposition index (Insulin) _0-30min._ | | | | | | | | |
| Baseline | 48 | 0.59±0.47  (0.45, 0.72) |  | 52 | 0.51±0.32  (0.42, 0.59) |  |  |  |
| After drug  washout | 47 | 0.63±0.43  (0.51, 0.76) |  | 53 | 0.62±0.36  (0.52, 0.72) |  |  |  |
| Change | 46 | 0.05±0.45  (-0.08, 0.18) |  | 51 | 0.12±0.38  (0.01, 0.22) |  |  |  |
| Ln disposition index (Insulin) _0-30min._ | | | | | | | | |
| Baseline | 48 | 0.02±0.36  (-0.09, 0.12) |  | 52 | -0.04±0.28  (-0.11, 0.04) |  | 0.05  (-0.08, 0.18) | 0.42 |
| After drug  washout | 47 | 0.07±0.33  (-0.03, 0.16) |  | 53 | 0.07±0.29  (-0.01, 0.15) |  | -0.01  (-0.13, 0.12) | 0.93 |
| Change | 46 | 0.06±0.32  (-0.04, 0.15) | 0.23 | 51 | 0.11±0.32  (0.02, 0.20) | 0.022 | -0.05  (0.18, 0.08) | 0.46 |
| Disposition index (CPR) _0-30min._ | | | | | | | | |
| Baseline | 48 | 0.07±0.10  (0.04, 0.10) |  | 52 | 0.06±0.03  (0.05, 0.07) |  |  |  |
| After drug  washout | 47 | 0.07±0.05  (0.05, 0.08) |  | 53 | 0.06±0.04  (0.06, 0.07) |  |  |  |
| Change | 46 | 0.002±0.08  (-0.02, 0.03) |  | 51 | 0.009±0.03  (-0.001, 0.02) |  |  |  |
| Ln disposition index (CPR) _0-30min._ | | | | | | | | |
| Baseline | 48 | -3.15±0.90  (-3.41, -2.89) |  | 52 | -3.08±0.69  (-3.27, -2.89) |  | -0.07  (-0.38, 0.25) | 0.67 |
| After drug  washout | 47 | -2.92±0.75  (-3.14, -2.70) |  | 53 | -2.89±0.60  (-3.06, -2.73) |  | -0.02  (-0.29, 0.25) | 0.87 |
| Change | 46 | 0.22±0.64  (0.03, 0.41) | 0.02 | 51 | 0.19±0.63  (0.01, 0.37) | 0.04 | 0.03  (-0.22, 0.29) | 0.79 |
| Insulinogenic index (Insulin) _0-120min._ | | | | | | | | |
| Baseline | 48 | 0.20±0.15  (0.16, 0.25) |  | 52 | 0.25±0.20  (0.19, 0.30) |  |  |  |
| After drug  washout | 47 | 0.23±0.19  (0.18, 0.29) |  | 53 | 0.31±0.26  (0.24, 0.39) |  |  |  |
| Change | 46 | 0.03±0.14  (-0.01, 0.07) |  | 51 | 0.07±0.15  (0.03, 0.11) |  |  |  |
| Ln insulinogenic index (Insulin) _0-120min._ | | | | | | | | |
| Baseline | 48 | -1.88±0.80  (-2.11, -1.65) |  | 52 | -1.72±0.82  (-1.95, -1.49) |  | 0.17  (-0.16, 0.49) | 0.31 |
| After drug  washout | 47 | -1.73±0.76  (-1.96, -1.51) |  | 53 | -1.48±0.83  (-1.71, -1.25) |  | 0.25  (-0.07, 0.57) | 0.12 |
| Change | 46 | 0.15±0.55  (-0.02, 0.31) | 0.08 | 51 | 0.25±0.49  (0.11, 0.39) | 0.0006 | 0.10  (-0.10, 0.31) | 0.32 |
| Insulinogenic index (CPR) _0-120min._ | | | | | | | | |
| Baseline | 49 | 0.03±0.02  (0.02, 0.04) |  | 54 | 0.04±0.02  (0.03, 0.04) |  |  |  |
| After drug  washout | 49 | 0.03±0.02  (0.03, 0.04) |  | 54 | 0.04±0.03  (0.03, 0.05) |  |  |  |
| Change | 49 | 0.004±0.02  -0.0007, 0.008) |  | 54 | 0.006±0.02  (0.001, 0.01) |  |  |  |
| Ln insulinogenic index (CPR) _0-120min._ | | | | | | | | |
| Baseline | 49 | -3.65±0.57  (-3.82, -3.49) |  | 54 | -3.52±0.57  (-3.67, -3.36) |  | -0.13  (-0.36, 0.09) | 0.24 |
| After drug  washout | 49 | -3.52±0.55  (-3.68, -3.36) |  | 54 | -3.35±0.57  (-3.51, -3.20) |  | -0.16  (-0.38, 0.06) | 0.14 |
| Change | 49 | 0.14±0.38  (0.03, 0.25) | 0.02 | 54 | 0.17±0.40  (0.06, 0.28) | 0.004 | -0.03  (-0.18, 0.12) | 0.70 |
| Matsuda index | | | | | | | | |
| Baseline | 48 | 5.53±4.87  (4.11, 6.94) |  | 52 | 5.08±2.95  (4.26, 5.90) |  |  |  |
| After drug  washout | 47 | 5.48±3.08  (4.57, 6.38) |  | 53 | 5.10±3.45  (4.15, 6.05) |  |  |  |
| Change | 46 | -0.14±3.38  (-1.14, 0.86) |  | 51 | -0.05±2.00  (-0.62, 0.51) |  |  |  |
| Ln Matsuda index | | | | | | | | |
| Baseline | 48 | 1.42±0.78  (1.19, 1.65) |  | 52 | 1.45±0.60  (1.29, 1.62) |  | -0.03  (-0.31, 0.24) | 0.81 |
| After drug  washout | 47 | 1.54±0.60  (1.36, 1.72) |  | 53 | 1.43±0.64  (1.26, 1.61) |  | 0.11  (-0.14, 0.35) | 0.39 |
| Change | 46 | 0.08±0.36  (-0.02, 0.19) | 0.12 | 51 | -0.04±0.31  (-0.13, 0.05) | 0.39 | 0.12  (-0.01, 0.26) | 0.08 |
| Serum proinsulin (pmol/L) | | | | | | | | |
| Baseline | 49 | 14.8±20.9  (8.8, 20.8) |  | 54 | 15.2±12.8  (11.8, 18.7) |  |  |  |
| After drug  washout | 49 | 12.4±13.0  (8.7, 16.1) |  | 54 | 17.9±15.7  (13.6, 22.1) |  |  |  |
| Change | 49 | -2.4±10.8  (-5.5, 0.7) |  | 54 | 2.6±6.6  (0.8, 4.4) |  |  |  |
| Ln serum proinsulin (pmol/L) | | | | | | | | |
| Baseline | 49 | 2.26±0.87  (2.01, 2.51) |  | 54 | 2.34±1.00  (2.07, 2.62) |  | -0.08  (-0.45, 0.29) | 0.66 |
| After drug  washout | 49 | 2.15±0.86  (1.91, 2.40) |  | 54 | 2.56±0.84  (2.33, 2.79) |  | -0.41  (-0.74, -0.07) | 0.02 |
| Change | 49 | -0.11±0.44  (-0.24, 0.01) | 0.08 | 54 | 0.21±0.61  (0.05, 0.38) | 0.01 | -0.32  (-0.53, -0.11) | 0.003 |
| Serum proinsulin/CPR ratio | | | | | | | | |
| Baseline | 49 | 6.21±4.35  (4.96, 7.45) |  | 54 | 6.64±3.58  (5.66, 7.61) |  |  |  |
| After drug  washout | 49 | 5.85±3.83  (4.75, 6.95) |  | 54 | 7.55±3.76  (6.52, 8.57) |  |  |  |
| Change | 49 | -0.36±2.30  (-1.02, 0.30) |  | 54 | 0.91±2.56  (0.21, 1.61) |  |  |  |
| Ln serum proinsulin/CPR ratio | | | | | | | | |
| Baseline | 49 | 1.63±0.63  (1.45, 1.81) |  | 54 | 1.70±0.75  (1.49, 1.90) |  | -0.06  (-0.33, 0.21) | 0.65 |
| After drug  washout | 49 | 1.56±0.68  (1.37, 1.76) |  | 54 | 1.90±0.51  (1.76, 2.04) |  | -0.34  (-0.57, -0.10) | 0.005 |
| Change | 49 | -0.07±0.35  (-0.17, 0.03) | 0.16 | 54 | 0.20±0.56  (0.05, 0.36) | 0.01 | -0.27  (-0.46, -0.09) | 0.004 |
| Serum Proinsulin/insulin ratio | | | | | | | | |
| Baseline | 49 | 1.93±1.62  (1.46, 2.39) |  | 54 | 2.18±1.73  (1.71, 2.65) |  |  |  |
| After drug  washout | 49 | 1.73±1.08  (1.42, 2.04) |  | 54 | 2.25±1.27  (1.90, 2.60) |  |  |  |
| Change | 49 | -0.20±0.85  (-0.45, 0.04) |  | 54 | 0.07±1.29  (-0.28, 0.42) |  |  |  |
| Ln serum proinsulin/insulin ratio | | | | | | | | |
| Baseline | 49 | 0.39±0.74  (0.17, 0.60) |  | 54 | 0.52±0.78  (0.30, 0.73) |  | -0.13  (-0.43, 0.17) | 0.38 |
| After drug  washout | 49 | 0.35±0.68  (0.15, 0.54) |  | 54 | 0.67±0.55  (0.52, 0.82) |  | -0.32  (-0.56, -0.08) | 0.01 |
| Change | 49 | -0.04±0.38  (-0.15, 0.07) | 0.48 | 54 | 0.15±0.66  (-0.03, 0.33) | 0.10 | -0.19  (-0.40, 0.02) | 0.08 |
| HbA1c (%) | | | | | | | | |
| Baseline | 49 | 7.6±0.4  (7.5, 7.7) |  | 54 | 7.5±0.5  (7.4, 7.7) |  | 0.1  (-0.1, 0.2) | 0.50 |
| 24weeks | 49 | 7.1±0.4  (7.0, 7.2) |  | 54 | 6.8±0.4  (6.7, 7.0) |  | 0.3  (0.1, 0.5) | <0.001 |
| Change | 49 | -0.5±0.5  (-0.6, -0.3) | <0.001 | 54 | -0.7±0.5  (-0.8, -0.5) | <0.001 | 0.2  (0.0, 0.4) | 0.02 |
| HbA1c (mmoL/moL) | | | | | | | | |
| Baseline | 49 | 59.3±4.9  (57.9, 60.7) |  | 54 | 58.6±5.4  (57.2, 60.1) |  | 0.7  (-1.3, 2.7) | 0.50 |
| 24weeks | 49 | 54.4±4.6  (53.1, 55.7) |  | 54 | 51.1±4.8  (49.8, 52.4) |  | 3.2  (1.4, 5.1) | <0.001 |
| Change | 49 | -5.0±5.0  (-6.4, -3.5) | <0.001 | 54 | -7.5±5.5  (-9.0, -6.0) | <0.001 | 2.5  (0.5, 4.6) | 0.02 |
| Fasting plasma glucose (mg/dL) | | | | | | | | |
| Baseline | 49 | 149.3±23.4  (142.6, 156.0) |  | 54 | 147.2±17.3  (142.4, 151.9) |  | 2.1  (-5.9, 10.1) | 0.60 |
| 24weeks | 49 | 126.3±16.7  (121.4, 131.1) |  | 54 | 134.3±17.4  (129.5, 139.1) |  | -8.0  (-14.7, -1.2) | 0.02 |
| Change at 24weeks | 49 | -23.0±19.2  (-28.5, -17.4) | <0.001 | 54 | -13.2±17.2  (-17.9, -8.4) | <0.001 | -9.8  (-17.0, -2.6) | 0.008 |
| After drug  washout | 49 | 138.6±22.5  (132.1, 145.0) |  | 54 | 140.6±21.1  (134.9, 146.4) |  | -2.0  (-10.6, 6.5) | 0.64 |
| Change at washout | 49 | -10.7±23.4  (-17.4, -4.0) | 0.002 | 54 | -6.6±20.8  (-12.2, -0.9) | 0.025 | -4.2  (-12.8, 4.5) | 0.34 |
| Body weight (kg) | | | | | | | | |
| Baseline | 49 | 69.9±12.3  (66.4, 73.4) |  | 54 | 71.9±19.1  (66.7, 77.1) |  | -1.9  (-8.3, 4.4) | 0.54 |
| 24weeks | 49 | 67.2±11.8  (63.9, 70.6) |  | 54 | 71.7±19.4  (66.4, 77.0) |  | -4.5  (-10.8, 1.9) | 0.16 |
| Change at 24weeks | 49 | -2.7±1.9  (-3.2, -2.1) | <0.001 | 54 | -0.1±1.9  (-0.7, 0.4) | 0.57 | -2.5  (-3.3, -1.8) | <0.001 |
| After drug  washout | 49 | 67.7±12.0  (64.2, 71.2) |  | 54 | 72.1±19.4  (66.8, 77.4) |  | -4.4  (-10.8, 2.0) | 0.18 |
| Change at washout | 49 | -2.2±1.9  (-2.8, -1.7) | <0.001 | 54 | 0.2±1.7  (-0.3, 0.7) | 0.40 | -2.4  (-3.1, -1.7) | <0.001 |
| Skeletal muscle (kg) | | | | | | | | |
| Baseline | 43 | 24.8±4.5  (23.4, 26.2) |  | 46 | 25.5±6.5  (23.6, 27.4) |  | -0.7  (-3.1, 1.7) | 0.57 |
| 24weeks | 41 | 24.2±4.4  (22.8, 25.6) |  | 45 | 25.5±6.7  (23.5, 27.5) |  | -1.3  (-3.8, 1.1) | 0.29 |
| Change at 24weeks | 41 | -0.6±0.7  (-0.8, -0.3) | <0.001 | 44 | 0.1±0.8  (-0.2, 0.3) | 0.47 | -0.6  (-1.0, -0.3) | <0.001 |
| Visceral fat area (cm^2^) | | | | | | | | |
| Baseline | 43 | 122.3±47.5  (107.7, 136.9) |  | 46 | 120.8±57.4  (103.7, 137.8) |  | 1.5  (-20.8, 23.8) | 0.89 |
| 24weeks | 41 | 115.2±48.1  (100.0, 130.3) |  | 45 | 119.5±56.4  (102.5, 136.4) |  | -4.3  (-26.9, 18.3) | 0.71 |
| Change at 24weeks | 41 | -8.1±10.4  (-11.4, -4.8) | <0.001 | 44 | -2.7±12.5  (-6.5, 1.1) | 0.15 | -5.3  (-10.3, -0.3) | 0.04 |
| Total ketone body (μmoL/L) | | | | | | | | |
| Baseline | 49 | 140.2±133.1  (101.9, 178.4) |  | 54 | 147.4±130.9  (111.6, 183.1) |  |  |  |
| 24weeks | 48 | 252.8±244.5  (181.8, 323.8) |  | 53 | 132.3±138.5  (94.2, 170.5) |  |  |  |
| Change at 24weeks | 48 | 114.8±192.7  (58.9, 170.8) |  | 53 | -16.9±166.2  (-62.7, 29.0) |  |  |  |
| After drug  washout | 49 | 103.9±85.0  (79.5, 128.3) |  | 54 | 137.9±115.0  (106.5, 169.3) |  |  |  |
| Change at washout | 49 | -36.3±144.7  (-77.9, 5.3) |  | 54 | -9.5±145.0  (-49.1, 30.1) |  |  |  |
| Ln Total ketone body (μmoL/L) | | | | | | | | |
| Baseline | 49 | 4.64±0.75  (4.42, 4.85) |  | 54 | 4.74±0.68  (4.56, 4.93) |  | -0.11  (-0.39, 0.17) | 0.45 |
| 24weeks | 48 | 5.12±0.97  (4.83, 5.40) |  | 53 | 4.58±0.73  (4.38, 4.78) |  | 0.54  (0.20, 0.87) | 0.002 |
| Change at 24weeks | 48 | 0.50±0.88  (0.24, 0.75) | <0.001 | 53 | -0.18±0.88  (-0.42, 0.06) | 0.14 | 0.68  (0.33, 1.03) | <0.001 |
| After drug  washout | 49 | 4.43±0.64  (4.25, 4.61) |  | 54 | 4.67±0.69  (4.48, 4.86) |  | -0.24  (-0.50, 0.02) | 0.07 |
| Change at washout | 49 | -0.21±0.73  (-0.41, 0.00) | 0.05 | 54 | -0.07±0.79  (-0.29, 0.14) | 0.50 | -0.13  (-0.43, 0.16) | 0.38 |
| Serum total adiponectin (μg/mL) | | | | | | | | |
| Baseline | 49 | 10.77±11.76  (7.39, 14.14) |  | 54 | 9.36±7.67  (7.26, 11.45) |  |  |  |
| After drug  washout | 49 | 12.14±11.52  (8.84, 15.45) |  | 54 | 9.53±7.31  (7.53, 11.53) |  |  |  |
| Change | 49 | 1.38±3.17  (0.47, 2.29) |  | 54 | 0.17±1.47  (-0.23, 0.58) |  |  |  |
| Ln serum total adiponectin (μg/mL) | | | | | | | | |
| Baseline | 49 | 2.07±0.72  (1.86, 2.27) |  | 54 | 2.04±0.58  (1.88, 2.20) |  | 0.02  (-0.23, 0.28) | 0.85 |
| After drug  washout | 49 | 2.22±0.70  (2.02, 2.42) |  | 54 | 2.06±0.60  (1.89, 2.22) |  | 0.16  (-0.09, 0.42) | 0.21 |
| Change | 49 | 0.15±0.17  (0.10, 0.20) | <0.001 | 54 | 0.01±0.14  (-0.02, 0.05) | 0.46 | 0.14  (0.08, 0.20) | <0.001 |
| Malondialdehyde (pmoL/mL) | | | | | | | | |
| Baseline | 49 | 268.6±129.6  (231.4, 305.8) |  | 54 | 217.8±166.6  (172.3, 263.3) |  |  |  |
| 24wks | 49 | 242.4±125.7  (206.3, 278.6) |  | 54 | 196.7±68.8  (177.9, 215.4) |  |  |  |
| Change at  24wks | 49 | -26.1±97.9  (-54.2, 2.0) |  | 54 | -21.1±145.6  (-60.9, 18.6) |  |  |  |
| Ln malondialdehyde (pmoL/mL) | | | | | | | | |
| Baseline | 49 | 5.48±0.49  (5.34, 5.62) |  | 54 | 5.26±0.44  (5.14, 5.38) |  | 0.22  (0.04, 0.40) | 0.02 |
| 24wks | 49 | 5.39±0.45  (5.26, 5.52) |  | 54 | 5.22±0.34  (5.13, 5.32) |  | 0.16  (0.01, 0.32) | 0.04 |
| Change at  24wks | 49 | -0.09±0.37  (-0.20, 0.02) | 0.09 | 54 | -0.04±0.33  (-0.13, 0.06) | 0.44 | -0.06  (-0.20, 0.08) | 0.41 |
| Skin advanced glycation end-products (AGEs) (AF) | | | | | | | | |
| Baseline | 31 | 2.39±0.55  (2.19, 2.60) |  | 29 | 2.32±0.47  (2.14, 2.50) |  | 0.08  (-0.19, 0.34) | 0.57 |
| 24wks | 30 | 2.43±0.45  (2.26, 2.60) |  | 30 | 2.38±0.42  (2.22, 2.54) |  | 0.05  (-0.18, 0.27) | 0.68 |
| Change at  24wks | 29 | 0.05±0.39  (-0.10, 0.20) | 0.51 | 27 | 0.04±0.36  (-0.10, 0.18) | 0.56 | 0.01  (-0.19, 0.21) | 0.94 |

Both values before and after logarithmic transformation were listed for parameters that did not show a normal distribution.
