# Supplementary material for: Comparison of protective effects of teneligliptin and luseogliflozin on pancreatic β-cell function: randomized, parallel-group, multicenter, open-label study (SECRETE-I study)
Source: Front Endocrinol (Lausanne). 2024 Oct 21;15:1412553. doi: 10.3389/fendo.2024.1412553 (PMC11532122; doi:10.3389/fendo.2024.1412553)
Supplement: Supplementary file 6 [file Table4.docx]

Supplementary Table 4. Comparison in adverse events between both groups

|  | No. of cases (%) | | Fisher  (*p*-value) |
| --- | --- | --- | --- |
|  | Luseogliflozin  group (n=55) | Teneligliptin  Group (n=56) |  |
| Any adverse events | 22 (40.0) | 9 (16.1) | 0.006 |
| Death | 0 (0.0) | 0 (0.0) | - |
| Any severe adverse events | 2 (3.6) | 1 (1.8) | 0.62 |
| Brain tumor | 1 (1.8) | 0 (0.0) | 0.50 |
| Lower extremity Arterio-Sclerosis Obliterans | 0 (0.0) | 1 (1.8) | 1.00 |
| Knee arthropathy | 1 (1.8) | 0 (0.0) | 0.50 |
| Polyuria | 2 (3.6) | 0 (0.0) | 0.24 |
| Genital itching | 4 (7.3) | 0 (0.0) | 0.06 |
| Balanitis | 1 (1.8) | 0 (0.0) | 0.50 |
| Urinary positive of yeast-like fungus | 1 (1.8) | 0 (0.0) | 0.50 |
| Cystitis | 2 (3.6) | 0 (0.0) | 0.24 |
| Constipation | 1 (1.8) | 1 (1.8) | 1.00 |
| Diarrhea | 1 (1.8) | 1 (1.8) | 1.00 |
| Liver damage | 1 (1.8) | 0 (0.0) | 0.50 |
| Epigastric discomfort | 1 (1.8) | 0 (0.0) | 0.50 |
| Hunger | 1 (1.8) | 0 (0.0) | 0.50 |
| Fatigue | 2 (3.6) | 0 (0.0) | 0.24 |
| Cavity | 2 (3.6) | 0 (0.0) | 0.24 |
| Coughing | 0 (0.0) | 2 (3.6) | 0.50 |
| Upper respiratory tract infection | 2 (3.6) | 4 (7.1) | 0.68 |
| Bronchitis | 1 (1.8) | 0 (0.0) | 0.50 |
| Allergic rhinitis | 1 (1.8) | 0 (0.0) | 0.50 |
| Hemorrhoid | 1 (1.8) | 0 (0.0) | 0.50 |
| Anemia | 1 (1.8) | 0 (0.0) | 0.50 |
| Dizziness | 2 (3.6) | 1 (1.8) | 0.62 |
| Shoulder discomfort | 1 (1.8) | 0 (0.0) | 0.50 |
| Rotator cuff tear | 1 (1.8) | 0 (0.0) | 0.50 |
| Cervicobrachial syndrome | 1 (1.8) | 0 (0.0) | 0.50 |
| Transverse process fracture of lumbar vertebra | 1 (1.8) | 0 (0.0) | 0.50 |
| Abnormal toe sensation | 0 (0.0) | 1 (1.8) | 1.00 |
| Plantar keratosis | 1 (1.8) | 0 (0.0) | 0.50 |
| Colorectal adenoma | 1 (1.8) | 0 (0.0) | 0.50 |
| Thyroid mass | 1 (1.8) | 0 (0.0) | 0.50 |
